# Functional Role of Resveratrol in Inducing Apoptosis in Breast Cancer Subtypes via Inhibition of Intracellular Fatty Acid Synthase

**DOI:** 10.3390/molecules30142891

**Published:** 2025-07-08

**Authors:** Ping Li, Yan Liang, Xiaofeng Ma

**Affiliations:** 1 University of Chinese Academy of Sciences, No. 19A Yuquan Road, Beijing 100049, China; liping111@mails.ucas.ac.cn; 2 Department of Medical College, Hunan Polytechnic of Environment and Biology, No. 165 Wangcheng Road, Hengyang 421001, China; 3 School of Kinesiology and Health, Capital University of Physical Education and Sports, No. 11 Beisanhuanxi Road, Beijing 100191, China

**Keywords:** resveratrol, fatty acid synthase, inhibitor, breast cancer cells, cell apoptosis

## Abstract

Fatty acid synthase (FASN) is frequently overexpressed in human breast cancer and has emerged as a potential therapeutic target. Resveratrol has been shown to inhibit FASN activity in vitro through both fast-reversible and slow-irreversible mechanisms. In this study, resveratrol reduced intracellular fatty acid levels by inhibiting FASN activity and downregulating its expression across various breast cancer subtypes, including SK-BR-3, MCF-7, and MDA-MB-231 cells. Knockdown of FASN via small interfering RNA (siRNA) further enhanced resveratrol-induced cytotoxicity. Resveratrol significantly suppressed cell viability and triggered apoptosis, as evidenced by increased cleavage of poly(ADP-ribose) polymerase (PARP) and disruption of Bcl-2 family protein balance. Furthermore, resveratrol inhibited key signaling pathways involved in cell proliferation and survival, notably FAK, AKT, and ERK1/2. FASN silencing by siRNA also modulated the activation states of these signaling proteins. Collectively, these findings support resveratrol as a promising anti-cancer candidate that induces apoptosis in diverse breast cancer subtypes via FASN inhibition.

## 1. Introduction

Breast cancer remains the most frequently diagnosed cancer and the leading cause of cancer-related mortality among women worldwide [1]. To address its heterogeneity and optimize therapeutic strategies, five intrinsic molecular subtypes of breast cancer—Luminal A, Luminal B, triple-negative (TNBC), HER-2-overexpressing, and normal breast-like—have been defined based on gene expression profiling [2]. These subtypes differ in their biological behaviors, prognostic outcomes, and responses to treatment modalities [3], highlighting the need for subtype-specific interventions and functional agents with broad-spectrum efficacy.

**Figure 1:**
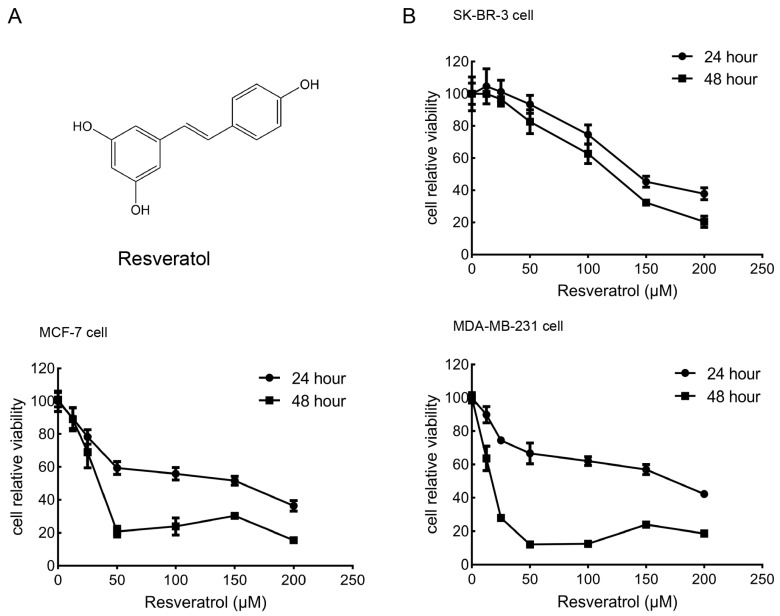
Effect of resveratrol on the viability of breast cancer cells. (**A**) Chemical structure of resveratrol. (**B**) The viability of SK-BR-3, MCF-7, and MDA-MB-231 cells treated with various concentrations of resveratrol (0, 12.5, 25, 50, 100, 150, 200 μM) for 24 h and 48 h as measured by a CCK-8 assay. The percentage of cell viability was calculated as the ratio of resveratrol-treated cells to control cells. Data represent the mean ± SD of three independent experiments.

Resveratrol (trans-3,5,4′-trihydroxystilbene, Figure 1A), a naturally occurring polyphenol abundantly found in functional foods such as grapes, berries, red wine, and peanuts, has attracted growing interest as a multi-targeted bioactive compound [4]. As a dietary phytochemical with demonstrated health-promoting properties, resveratrol has been associated with antioxidant, anti-inflammatory, anti-aging, cardioprotective, neuroprotective, and metabolic regulatory effects, rendering it a promising agent in the field of functional foods and preventive nutrition [5]. Importantly, its potential anti-cancer effects have been extensively studied in vitro and in vivo. Resveratrol inhibits cancer initiation, promotion, and progression in various animal models [6,7], and exhibits cytotoxic effects on multiple breast cancer cell lines through mechanisms involving cell cycle arrest, apoptosis induction, suppression of invasion and metastasis, epigenetic regulation, and increased chemosensitivity [8,9].

Recent studies have emphasized that the biological effects of resveratrol extend beyond traditional signaling pathways and involve direct modulation of tumor metabolism, including lipid biosynthesis and energy homeostasis [10]. Altered metabolic reprogramming, particularly increased lipogenesis, is a hallmark of cancer cells, supporting their proliferation and survival within the hostile tumor microenvironment [11]. In this context, fatty acid synthase (FASN), a key enzyme catalyzing the de novo synthesis of long-chain fatty acids from acetyl-CoA, malonyl-CoA, and NADPH, emerges as a critical metabolic target [12,13,14]. FASN is frequently overexpressed in breast cancer and correlates with poor prognosis, aggressive phenotype, and resistance to therapy [15]. Consequently, FASN inhibition represents a rational strategy for breast cancer treatment, especially in metabolically active subtypes such as TNBC.

Given its dietary origin and broad-spectrum antitumor activities, resveratrol exemplifies the concept of a functional food-derived compound with chemopreventive and therapeutic potential. However, despite growing evidence of its benefits, the subtype-specific effects of resveratrol and its underlying metabolic mechanisms—particularly via FASN inhibition—remain insufficiently understood [16]. Exploring how resveratrol modulates lipid metabolism to induce apoptosis across different breast cancer subtypes may offer novel insights into its role as a functional agent and support its application in integrative cancer prevention strategies. Although previous studies have reported that resveratrol downregulates FASN expression in HER-2-positive and FASN-overexpressing breast cancer SK-BR-3 cell line, the full scope of its anti-cancer potential remains to be clarified [17]. In particular, the question of whether this regulatory effect extends beyond a single cell line has not been systematically investigated. In this study, we explored the broader applicability of resveratrol across multiple molecular subtypes of breast cancer [8]. Our findings demonstrate that resveratrol not only suppresses FASN expression but also significantly reduces intracellular FASN enzymatic activity in a variety of breast cancer cell lines, highlighting a more universal mechanism of action. These results suggest that resveratrol, a functional food-derived compound, could serve as a multi-targeted therapeutic candidate for diverse breast cancer subtypes via modulation of lipid metabolism.

## 2. Results

### 2.1. Effects of Resveratrol on the Viability of Breast Cancer Cells

To assess the cytotoxic impact of resveratrol on breast cancer cell lines, SK-BR-3, MCF-7, and MDA-MB-231 cells were treated with a gradient of resveratrol concentrations (0, 12.5, 25, 50, 100, 150, 200 μM) for durations of 24 and 48 h. As illustrated in Figure 1B, resveratrol significantly suppressed cell viability in a dose- and time-dependent manner across all three cell types. The half-maximal inhibitory concentrations (IC_50_) at 24 h were 151.4 μM for SK-BR-3, 102.6 μM for MCF-7, and 125.2 μM for MDA-MB-231, whereas at 48 h, the IC_50_ values decreased to 114.2 μM, 38.3 μM, and 14.1 μM, respectively. Despite slight differences in sensitivity, all three cell lines exhibited a consistent downward trend in viability following resveratrol exposure. These findings suggest that resveratrol exerts a cytotoxic effect and inhibits proliferation in human breast cancer cells.

### 2.2. Resveratrol Induces Breast Cancer Cells Apoptosis

**Figure 2:**
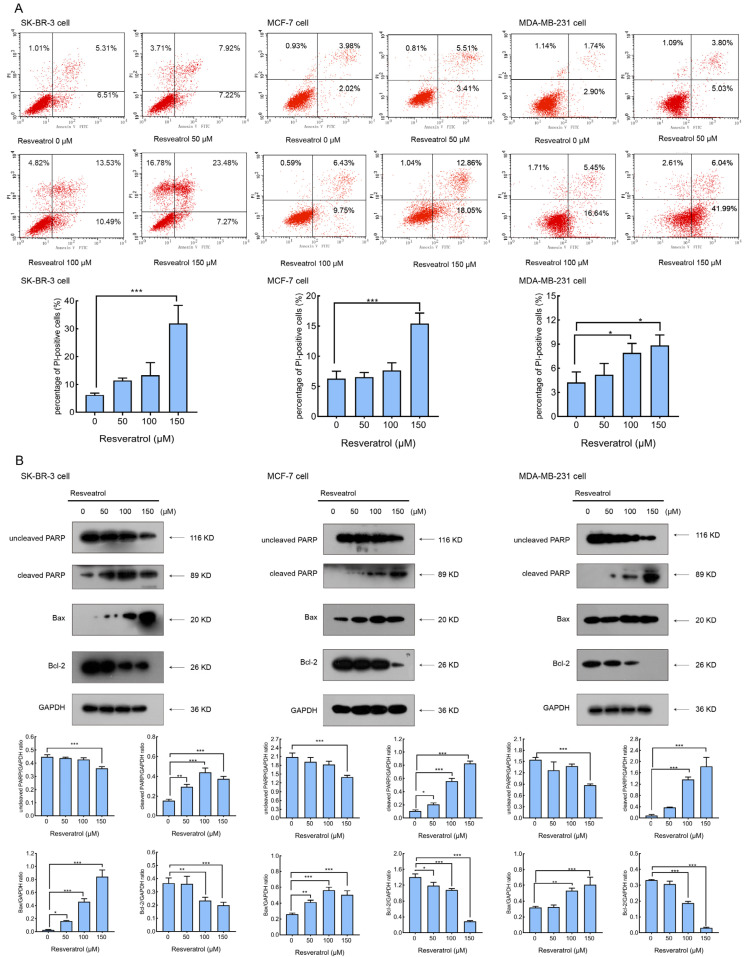
Resveratrol-induced apoptosis in breast cancer cells. (**A**) SK-BR-3, MCF-7, and MDA-MB-231 cells were treated with 0, 50, 100, 150 μM resveratrol for 24 h, then stained with Annexin V/PI and analyzed by flow cytometry. The percentage of cells in each quadrant is indicated (bottom left: viable; top left: necrotic; bottom right: early apoptotic; top right: late apoptotic). The bar graphs below show the quantification of PI-positive (necrotic and late apoptotic) cells at each resveratrol concentration. (**B**) After treatment as described above, the expression levels of PARP, cleaved PARP, Bax, and Bcl-2 were analyzed by Western blotting with GAPDH as a loading control. Blots shown are representative of at least two independent experiments. Densitometric analysis of protein bands was performed using ImageJ version 1.53c (National Institutes of Health, USA), and quantification is presented in the bar graphs below each blot. Data are shown as the mean ± SD from at least three independent experiments. Statistical significance was determined using one-way ANOVA followed by Tukey’s post hoc test. * *p* < 0.05, ** *p* < 0.01, and *** *p* < 0.001.

To determine whether the resveratrol-induced decline in cell viability was associated with apoptosis, we assessed apoptotic cell death in the three breast cancer cell lines after 24 h of treatment using Annexin V-FITC and propidium iodide (PI) staining followed by flow cytometry. As shown in Figure 2A, resveratrol treatment led to a notable, concentration-dependent increase in both early and late apoptotic cell populations, suggesting that the observed growth inhibition involved apoptotic pathways. Notably, quantification of PI-positive cells revealed that treatment with 150 μM resveratrol led to a significant increase in the proportion of necrotic (Annexin V^−^/PI^+^) cells in the SK-BR-3 cell line compared with MCF-7 and MDA-MB-231 cells (Figure 2A, bar graphs). Specifically, the percentage of PI-positive necrotic cells in SK-BR-3 cells increased sharply to 23.48% at 150 μM, whereas the increases in MCF-7 and MDA-MB-231 cells were relatively modest (9.75% and 16.64%, respectively). Among the three cell lines, MDA-MB-231 exhibited the lowest overall PI-positive rate across all concentrations. This pronounced induction of necrosis in SK-BR-3 cells may be attributed to their intrinsic cellular characteristics, including a slower proliferation rate and lower baseline viability compared to the other two lines, as previously reported [18,19]. SK-BR-3 cells, which are HER-2-positive and less proliferative, may be more vulnerable to necrotic cell death under high-dose resveratrol treatment due to their limited capacity for stress adaptation, whereas the more rapidly proliferating MDA-MB-231 and MCF-7 cells display greater resistance to necrosis under similar conditions. Additionally, Western blot analysis was performed to evaluate the expression levels of key apoptosis-related proteins, including cleaved PARP, Bax, and Bcl-2. As illustrated in Figure 2B, resveratrol dose-dependently enhanced the accumulation of cleaved PARP (89 kDa), upregulated the pro-apoptotic protein Bax, and downregulated the anti-apoptotic protein Bcl-2. These results confirm that apoptosis contributes to resveratrol-mediated cytotoxicity in breast cancer cells.

### 2.3. Resveratrol Inhibits FASN Activity and Down-Regulated FASN Expression in Breast Cancer Cells

**Figure 3:**
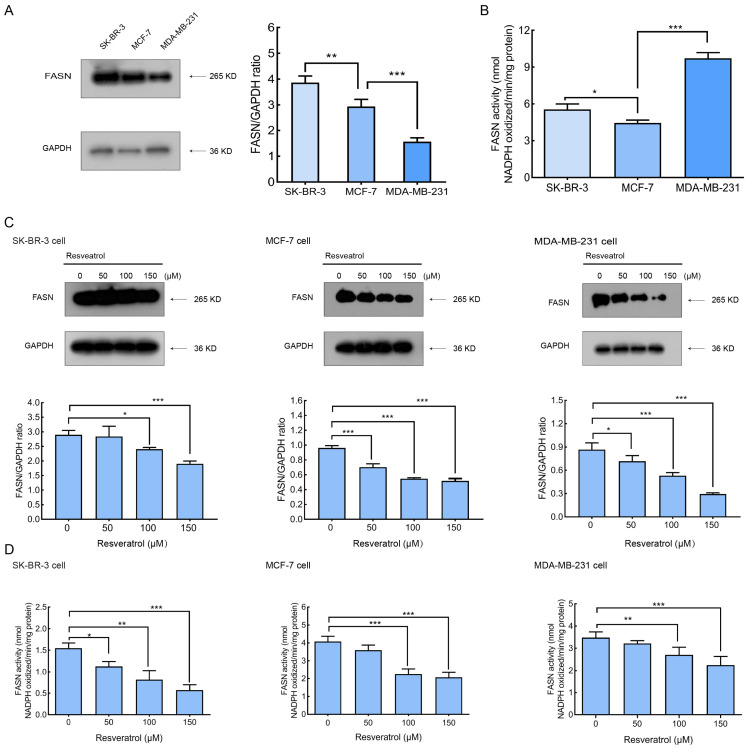
Resveratrol down-regulated FASN expression and inhibited intracellular FASN activity. (**A**) The expression levels of FASN in SK-BR-3, MCF-7, and MDA-MB-231 cells were examined by Western blotting. Representative blots and densitometric quantification (FASN/GAPDH ratio) from three independent experiments are shown. (**B**) Intracellular FASN activity in breast cancer cells was measured by spectrophotometrically monitoring the oxidation of NADPH at 340 nm. (**C**) Treatment with various concentrations of resveratrol for 24 h in breast cancer cells inhibited FASN expression, as determined by Western blotting. Densitometric analysis of protein bands was performed using ImageJ software (version 1.53c, National Institutes of Health, USA). (**D**) Resveratrol-induced inhibition of intracellular FASN activity in breast cancer cells; FASN activity was measured by monitoring the decrease in NADPH at 340 nm. Data represent the mean ± SD of three independent experiments. Statistical analysis was performed using one-way ANOVA followed by Tukey’s post hoc test. * *p* < 0.05 vs. control (0 μM); ** *p* < 0.01 vs. control; *** *p* < 0.001 vs. control.

We initially evaluated the FASN expression profiles in SK-BR-3, MCF-7, and MDA-MB-231 breast cancer cell lines. In agreement with prior findings, SK-BR-3 cells exhibited high FASN protein levels, while MCF-7 and MDA-MB-231 cells displayed intermediate and low levels, respectively (Figure 3A). Notably, the enzymatic activity of FASN did not correlate directly with its expression levels. FASN activity in MDA-MB-231 cells, which expressed the lowest FASN protein, was approximately 1.5-fold higher than in SK-BR-3 cells, which highly overexpressed FASN. Meanwhile, MCF-7 cells with moderate FASN expression demonstrated the lowest enzymatic activity among the three lines (Figure 3B). This observation deviated from earlier reports [20].

It is well established that upregulation of FASN is a frequent molecular event in breast cancer. Considering that resveratrol was previously shown to inhibit purified chicken FASN with an IC_50_ of 11.1 μg/mL in vitro, we further explored whether resveratrol modulates FASN expression and intracellular activity in breast cancer cells. As shown in Figure 3C, resveratrol treatment for 24 h led to a concentration-dependent reduction in FASN protein levels in both MCF-7 and MDA-MB-231 cells. However, FASN expression in SK-BR-3 cells was only marginally affected. Subsequent analysis of intracellular FASN enzymatic activity revealed a marked decrease in all three breast cancer cell lines following resveratrol exposure for 24 h (Figure 3D).

### 2.4. Silencing FASN via siRNA Potentiates the Cytotoxic Effect of Resveratrol in Breast Cancer Cells

**Figure 4:**
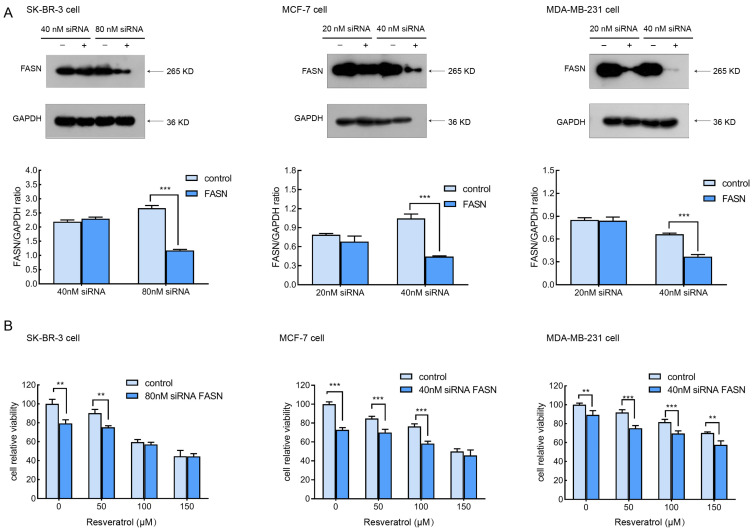
Resveratrol showed more potent cytotoxicity in FASN knockdown breast cancer cells. (**A**) SK-BR-3, MCF-7, and MDA-MB-231 cells were transfected with scrambled siRNA or siRNA targeting FASN for 72 h; the expression levels of FASN were analyzed by Western blotting, with GAPDH as a loading control. Densitometric analysis of FASN bands was quantified and normalized to GAPDH. (**B**) After transfection with FASN-targeting siRNA or control siRNA for 72 h, cells were treated with resveratrol for 24 h, and cell viability was determined by CCK-8 assay. The percentage of cell viability was calculated as the ratio of resveratrol-treated cells to control cells. Data are presented as the mean ± SD from three independent biological replicates (*n* = 3). Statistical analysis was performed using two-way ANOVA followed by Bonferroni’s post hoc test followed for multiple comparisons. ** *p* < 0.01, and *** *p* < 0.001 versus the indicated controls.

To further investigate the role of FASN in modulating resveratrol sensitivity, SK-BR-3, MCF-7, and MDA-MB-231 cells were transfected with FASN-specific siRNA to effectively reduce FASN expression. A scrambled siRNA served as the negative control. As illustrated in Figure 4A, treatment with 40 nM FASN siRNA in MCF-7 and MDA-MB-231 cells, or 80 nM in SK-BR-3 cells, led to a pronounced knockdown of FASN protein levels compared to control cells. To determine whether FASN depletion influences resveratrol-induced cell death, cells were collected 72 h post-transfection, seeded into 96-well plates, and treated with varying concentrations of resveratrol. Cell viability was subsequently assessed. As shown in Figure 4B, silencing FASN significantly amplified resveratrol’s cytotoxic impact relative to control siRNA-transfected cells. This outcome contrasts with previous findings regarding the FASN inhibitor C75, which was reported to mitigate cytotoxic effects following FASN knockdown [21].

### 2.5. Resveratrol or siRNA-Targeted FASN Reduces the Amount of Intracellular Fatty Acid

**Figure 5:**
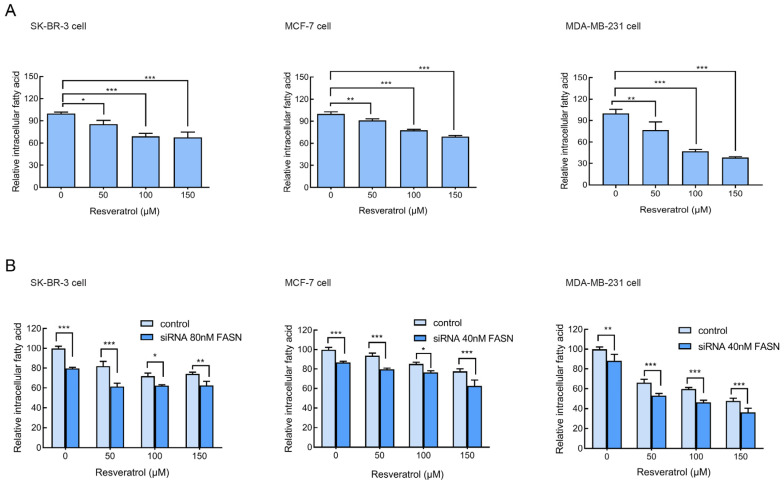
Resveratrol or siRNA-targeted FASN reduced intracellular fatty acid levels in breast cancer cells. (**A**) SK-BR-3, MCF-7, and MDA-MB-231 cells were treated with increasing concentrations of resveratrol (0, 50, 100, and 150 μM) for 24 h. Intracellular fatty acid content was measured using a Free Fatty Acid Quantification Kit. (**B**) Cells were transfected with siRNA targeting FASN or control siRNA for 72 h, followed by treatment with resveratrol (0, 50, 100, or 150 μM) for 24 h. Intracellular fatty acid levels were subsequently determined. Data are presented as the mean ± SD from three independent biological replicates (*n* = 3). Statistical analysis was performed using one-way ANOVA by Tukey’s post hoc test or two-way ANOVA by Bonferroni’s post hoc test followed for multiple comparisons. * *p* < 0.05, ** *p* < 0.01, and *** *p* < 0.001 indicate statistical significance compared to the indicated controls.

FASN is involved in the production of phospholipids required for cancer cells. Inhibition of FASN could reduce both the amount of fatty acid and phospholipids [21,22]. The results of intracellular fatty acid quantification showed that treating with 50 μM resveratrol for 24 h reduced the amount of intracellular fatty acid in breast cancer cells evidently (Figure 5A). We further proved that a combination of resveratrol and FASN siRNA treatment decreased the amount of intracellular fatty acid compared with resveratrol treatment alone in breast cancer cells (Figure 5B).

### 2.6. Resveratrol and FASN Knockdown Modulate Multiple Signaling Cascades Linked to Breast Cancer Cell Growth and Survival

**Figure 6:**
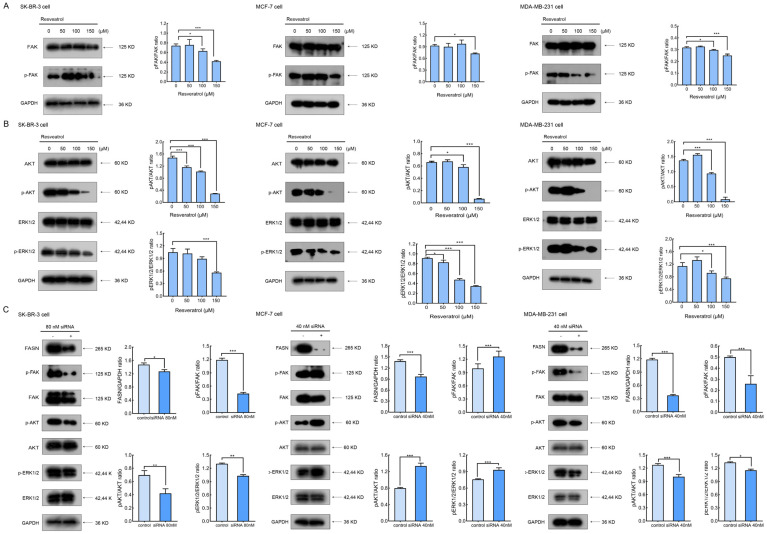
Resveratrol or siRNA-mediated FASN attenuation of signaling pathways associated with breast cancer cell proliferation and survival. (**A**) SK-BR-3, MCF-7, and MDA-MB-231 cells were treated with various concentrations of resveratrol (0, 50, 100, and 150 μM) for 24 h, and phosphorylation of FAK was analyzed by Western blotting. (**B**) Phosphorylation of AKT and ERK1/2 was assessed in cells treated with increasing concentrations of resveratrol for 24 h, as determined by Western blotting. (**C**) Breast cancer cells were transfected with scrambled control siRNA or siRNA targeting FASN for 72 h. The phosphorylation levels of FAK, AKT, and ERK1/2 were determined by Western blotting to confirm the regulatory effect of FASN knockdown. GAPDH served as a loading control. Densitometric analysis was performed and data are shown as the mean ± SD from three independent biological replicates (*n* = 3). Statistical significance was assessed using one-way ANOVA with Tukey’s post hoc test. * *p* < 0.05, ** *p* < 0.01, and *** *p* < 0.001.

The suppression of FASN activity, either through resveratrol treatment or siRNA-mediated gene silencing, was associated with alterations in signaling pathways crucial for the proliferation and survival of breast cancer cells. In particular, focal adhesion kinase (FAK) signaling plays a pivotal role in tumor development, cell proliferation, invasion, and metastatic progression [23,24,25]. This prompted an investigation into the impact of resveratrol on the phosphorylation of FAK at tyrosine 397 (p-FAK), a key regulatory site. Treatment with 150 μM resveratrol for 24 h resulted in a significant decline in p-FAK levels (Figure 6A), indicating downregulation of FAK-dependent signaling.

We further examined the phosphorylation status of ERK1/2 and AKT following 24 h resveratrol exposure (0–150 μM). The data demonstrated a dose-dependent suppression of the phosphorylated forms, p-ERK1/2 and p-AKT, without appreciable change in total ERK1/2 or AKT protein expression (Figure 6B).

To reinforce the role of FASN in regulating these pathways, we assessed the effects of FASN silencing via siRNA. In SK-BR-3 and MDA-MB-231 cells, FASN depletion caused a noticeable reduction in p-FAK, p-AKT, and p-ERK1/2, further supporting the pro-apoptotic consequences of targeting FASN. Conversely, in MCF-7 cells, FASN knockdown unexpectedly led to upregulation of these phosphorylated signaling proteins (Figure 6C), suggesting a cell line-dependent regulatory mechanism. These findings imply that siRNA-based FASN suppression may impair critical survival signaling in breast cancer cells.

## 3. Discussion

Breast cancer originates from normal mammary epithelial cells and is classified into five main molecular subtypes: Luminal A, Luminal B, HER-2 overexpression, triple-negative breast cancer, and normal-like breast cancer [26]. Our findings, consistent with previous reports [27], indicated a gradient of FASN expression across breast cancer cell lines, with SK-BR-3 exhibiting high levels, MCF-7 moderate, and MDA-MB-231 very low expression. This heterogeneity reflects distinct metabolic phenotypes among breast cancer subtypes, particularly regarding lipid metabolism [28]. Targeting enzymes involved in these metabolic pathways, such as FASN, has emerged as a promising therapeutic strategy in breast cancer treatment [29]. Consistent with earlier findings showing that resveratrol suppresses FASN expression in SK-BR-3 cells, our study confirms this effect and further extends the understanding of its anti-cancer action. Notably, we observed that resveratrol exerts similar FASN-targeting effects across multiple breast cancer cell lines representing distinct molecular subtypes. Moreover, our results provide new evidence that resveratrol not only downregulates FASN at the transcriptional and translational levels but also significantly inhibits its intracellular enzymatic activity. This dual action—on both FASN expression and catalytic function—may contribute to the broad-spectrum pro-apoptotic effects observed in various breast cancer models.

The differential expression and activity of FASN between malignant and normal cells underline its potential as a molecular target for anti-cancer drug development [18,30,31,32,33]. Given the frequent overexpression of FASN in diverse human tumors [12], interventions aimed at disrupting endogenous fatty acid synthesis by FASN inhibition hold clinical value. Our prior studies and those of others have documented that FASN inhibitors induce apoptosis selectively in cancer cells with elevated FASN expression [34,35]. The current work extends these findings by demonstrating that resveratrol, a natural FASN inhibitor, suppresses both FASN expression and enzymatic activity, leading to apoptotic cell death across various breast cancer subtypes, independent of baseline FASN expression levels.

While the antitumor effects of resveratrol in breast cancer have been described [7,36], comparative analyses across molecular subtypes characterized by distinct FASN expression remain limited. Our data confirm that resveratrol exerts cytotoxic effects by disrupting cellular homeostasis, as evidenced by increased PARP cleavage, upregulation of the pro-apoptotic protein Bax, and downregulation of anti-apoptotic Bcl-2 across SK-BR-3, MCF-7, and MDA-MB-231 cells. These results further reinforce resveratrol’s role as a potent anti-cancer agent.

Mechanistically, we hypothesized that FASN serves as a key target for resveratrol-induced apoptosis in breast cancer cells. Indeed, our study is the first to report a significant decrease in intracellular FASN expression and activity following resveratrol treatment in multiple breast cancer subtypes, with no notable differences observed among the subtypes in this regard. Cancer cells often exhibit FASN hyperactivity to meet increased demands for long-chain fatty acids, which are incorporated into membrane lipid rafts essential for modulating growth factor-mediated oncogenic pathways involved in cell survival, proliferation, migration, and invasion [18,37]. Overexpression of FASN likely confers resistance to apoptosis [29]; therefore, its inhibition disrupts phospholipid synthesis, suppresses tumor proliferation, and triggers programmed cell death. We also demonstrated that resveratrol reduces intracellular free fatty acid levels, implicating fatty acid biosynthesis as critical for its cytotoxic effects. This contrasts with findings in rat hepatocytes, where resveratrol inhibited fatty acid and triacylglycerol synthesis by reducing acetyl-CoA carboxylase (ACC) activity without affecting FASN [38], highlighting cancer-specific effects.

Previous studies have linked the cytotoxic sensitivity of breast cancer cells to classical FASN inhibitors like cerulenin and C75 with basal FASN expression levels [18,39]. However, such correlations had not been explored with resveratrol until now. Our data reveal that combining FASN siRNA with resveratrol significantly enhances cytotoxicity compared to resveratrol alone, paralleling observations with C75 [18]. These findings suggest that FASN acts as an anti-apoptotic factor in breast cancer cells, as downregulation by siRNA elevates apoptosis.

We further investigated the impact of FASN inhibition on lipid raft-mediated cell signaling. Focal adhesion kinase (FAK), an intracellular tyrosine kinase, plays a pivotal role in transducing extracellular growth factor and integrin signals through PI3K and MAPK pathways. Elevated expression of both FASN and FAK protects cancer cells from apoptosis, enhancing their survival [40]. FAK promotes tumor cell adhesion and functions as a survival signal, inhibiting apoptosis. Prior studies have demonstrated that both resveratrol treatment and FASN silencing reduce FAK phosphorylation [41,42]. Here, we confirmed that resveratrol decreased FAK activation in all three breast cancer cell lines. FAK activation correlates with growth factor signaling and estrogen receptor activity in breast cancer cells [43]. Inhibition of FAK has shown synergistic apoptotic effects with EGF receptor blockade in breast cancer [44], suggesting that disrupting phospholipid synthesis via FASN inhibition impairs EGFR localization, attenuates FAK-associated signaling, and induces apoptosis.

The PI3K/AKT and MAPK/ERK1/2 pathways transmit growth factor signals from the membrane to the nucleus and regulate proliferation and survival [45,46]. These pathways also modulate FASN expression through upstream signals, including EGFR and steroid hormone receptors [47]. Our results demonstrated that resveratrol markedly decreased phosphorylated ERK1/2 and AKT levels in all tested breast cancer cells, consistent with a reduction in membrane phospholipid synthesis and subsequent disruption of lipid raft assembly and EGFR localization [48]. Interestingly, siRNA-mediated FASN knockdown decreased p-FAK, p-AKT, and p-ERK1/2 in SK-BR-3 and MDA-MB-231 cells but paradoxically increased these phosphorylated proteins in MCF-7 cells. This differential response may reflect inherent cellular characteristics of MCF-7, where fatty acid synthesis inhibition triggers compensatory survival signaling to protect against apoptosis, similar to chemotherapy-induced anti-apoptotic mechanisms [39].

Some limitations of this study merit consideration. An intriguing attenuation of resveratrol efficacy was observed in transfected versus non-transfected cells across all tested lines. In SK-BR-3, intracellular fatty acid content increased from ~30% to 70% under identical treatment, likely due to competitive uptake inhibition by cationic transfection lipids and activation of compensatory survival pathways (e.g., PI3K/Akt) during transfection [49,50]. This metabolic interference was particularly pronounced in FASN-overexpressing subtypes, suggesting transient reductions in therapeutic efficacy [51,52]. These findings underscore critical methodological considerations for combining small molecules with genetic manipulation techniques [53].

Our in vitro experiments employed resveratrol concentrations of 50–150 µM; however, pharmacokinetic data from humans and animal studies demonstrate that such levels are not reached in vivo. Oral resveratrol is rapidly absorbed (≥70% of a 25 mg dose) but undergoes extensive first-pass metabolism, resulting in peak plasma concentrations of unchanged resveratrol of approximately 0.5–2 µM and typically <5 ng/mL (≈0.02 µM), with metabolic conjugates reaching 2–6 µM [54,55,56]. Rodent pharmacokinetics likewise show transient free-resveratrol peaks of ~2–3 µM after intravenous or oral dosing [57]. Consequently, physiologically achievable systemic concentrations are far below the 50–150 µM used in our assays, highlighting a major limitation in translating these findings in vivo and underscoring the need for advanced delivery systems or higher dosing strategies. A critical question for therapeutic targeting of FASN is the selectivity of its inhibition. Future research should determine whether resveratrol similarly inhibits FASN activity in normal cells, which typically express low FASN levels, to assess potential off-target effects. Moreover, elucidating how FASN inhibition contributes to tumor growth suppression in vivo is essential, warranting studies using tumor-bearing and FASN knockdown mouse models. Given FAK’s role in invasion and metastasis, further investigations are needed to clarify the relationship between FASN inhibition and metastatic behavior observed here.

## 4. Materials and Methods

### 4.1. Reagents and Antibodies

Acetyl-CoA, malonyl-CoA, NADPH, dimethyl sulfoxide (DMSO), and resveratrol were purchased from Sigma (St. Louis, MO, USA). Gibco (Beijing, China) supplied Dulbecco’s modified Eagle’s medium (DMEM), while fetal bovine serum (FBS) was obtained from Sijiqing Biological Engineering Materials (Zhejiang, China). Antibodies specific for FASN, Bax, Bcl-2, PARP, FAK, phosphor-FAK (Tyr397), AKT, phosphor-AKT (Ser473), ERK1/2, phosphor-ERK1/2 (Thr202/Tyr204), and GAPDH were sourced from Cell Signaling Technology (Denvers, MA, USA). All other chemicals were of high purity and acquired from commercial suppliers.

### 4.2. Cell Culture

Human breast cancer cell lines SK-BR-3, MCF-7, and MDA-MB-231 cells were purchased from Shanghai Institute of Cell Biology (Shanghai, China). Cells were maintained in DMEM medium supplemented with 10% FBS at 37 °C in a humidified atmosphere of 5% CO_2_ and 95% air.

### 4.3. Cell Viability Assay

Cell viability was determined using the Cell Counting Kit-8 (CCK-8; Dojindo, Japan) according to a previously described protocol [58]. Briefly, cells were seeded in 96-well plates at 1 × 10^4^ cells/well and cultured overnight. Following removal of the initial medium, cells were treated with fresh medium containing resveratrol at varying concentrations (five replicates per group) for 24 or 48 h, using DMSO as a vehicle control. Subsequently, each well was supplemented with 100 μL fresh medium and 10 μL CCK-8 reagent, followed by 1-h incubation at 37 °C. Absorbance at 450 nm was measured using a Multiskan MK3 microplate reader. Data represent the mean ± SD from three independent experiments.

### 4.4. Cell Apoptosis Assay

Apoptosis was assessed using an Annexin V-FITC Detection Kit (Becton Dickinson, Franklin Lakes, NJ, USA). Briefly, treated cells were harvested, washed twice with ice-cold PBS, and resuspended in 1× binding buffer (1 × 10^6^ cells/mL). A 500 μL aliquot of the suspension was mixed with 5 μL Annexin V-FITC and 10 μL propidium iodide (PI) in a flow cytometry tube. Following 15-min dark incubation at room temperature, samples were analyzed on a FACScalibur flow cytometer (Becton Dickinson). Annexin V^+^/PI^−^ cells were defined as early apoptotic, while Annexin V^+^/PI^+^ populations indicated late apoptosis.

### 4.5. Intracellular FASN Activity Assay

The intracellular FASN activity was measured by detecting the absorption at 340 nm to monitor the decrease in NADPH and was expressed in nmoles NADPH oxidized min^−1^ mg protein^−1^, as previously described [34].

### 4.6. Western Blotting Analysis

Post-treatment, cells were washed twice with ice-cold PBS and lysed on ice for 15 min in RIPA buffer containing protease inhibitors (Roche). Lysates were centrifuged at 14,000× *g* for 15 min, and supernatant protein concentrations determined using a BCA assay kit (Pierce). Equal protein amounts were denatured in Laemmli buffer at 95 °C for 5 min, then resolved by 8–12% SDS-PAGE and electrotransferred to PVDF membranes. After blocking nonspecific sites with 5% non-fat milk/TBST (4 h, at room temperature), membranes were incubated overnight at 4 °C with primary antibodies diluted in blocking buffer. Following three 10-min TBST washes, membranes were incubated with HRP-conjugated secondary antibodies (1 h, at room temperature) and visualized using Super Signal West Pico ECL substrate (Thermo Fisher Scientific: Suzhou, China). GAPDH served as the loading control. The density of the bands was measured by Image software.

### 4.7. Small Interference RNA Transfection

Breast cancer cells underwent transient transfection with FASN-targeting siRNA (sequence: 5′-TGGAGCGTATCTGTGAGAATT-3′), as previously established [59]. A scrambled siRNA duplex (5′-UUCUCCGAACGUGUCACGUTT-3′) (Invitrogen: Carlsbad, CA, USA) served as the negative control. Using Lipofectamine 2000 (Invitrogen), cells in 60-mm dishes were transfected for 6 h with 20–80 nM of either FASN siRNA or scrambled siRNA, following manufacturer protocols. Cells were harvested 48 h post-transfection for downstream analysis.

### 4.8. Intracellular Free Fatty Acid Quantification

Intracellular free fatty acid was measured using the Fatty Acid Quantification Kit (BioVision: Zurich, Switzerland). Briefly, cells were treated as indicated, then lysed and processed according to the kit’s protocol to determine fatty acid content, normalized to protein concentration.

### 4.9. Statistical Analysis

All quantitative data are presented as the mean ± standard deviation (SD) from at least three independent biological replicates. Statistical analyses were performed using GraphPad Prism version 8.0 (GraphPad Software, San Diego, CA, USA). For comparisons involving more than two groups (e.g., different concentrations of resveratrol, or factorial experiments combining siRNA and resveratrol treatment), one-way or two-way analysis of variance (ANOVA) was applied as appropriate, followed by Tukey’s or Bonferroni’s post hoc multiple comparison tests. For comparisons between two groups, the unpaired Student’s *t* test was used. Differences were considered statistically significant at *p* < 0.05. The specific statistical tests applied to each experiment are indicated in the corresponding figure legends.

## 5. Conclusions

**Figure 7:**
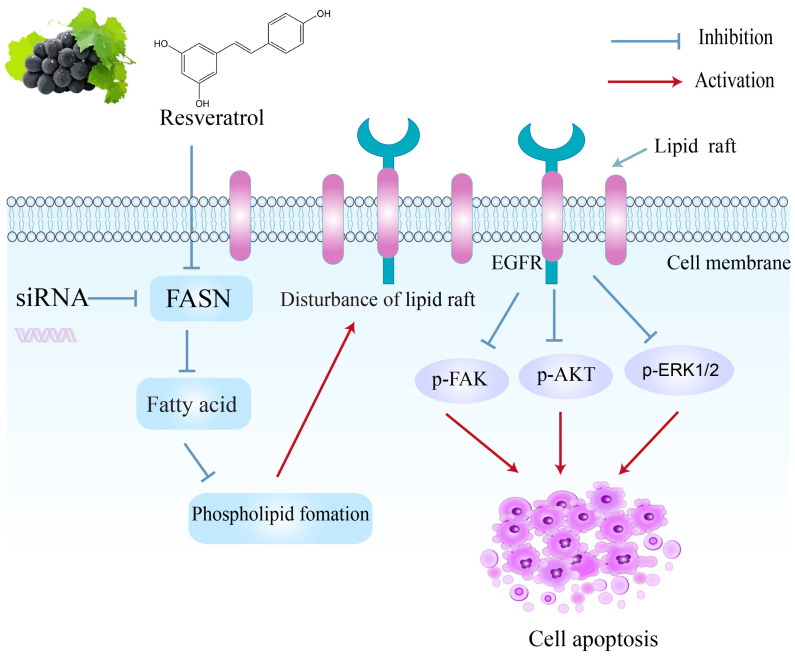
The proposed mechanism of resveratrol-induced apoptosis via inhibition of FASN.

Our current study demonstrated that resveratrol, as a highly active FASN inhibitor, inhibited both the intracellular FASN activity and FASN expression, and thus induced apoptosis among the three molecular subtypes of breast cancer cell lines. Blockade of fatty acid synthesis by resveratrol may impair the proper localization of EGFR to the cell membrane in breast cancer cells, which attenuated several signaling pathways associated with breast cancer cell proliferation and survival (Figure 7). Resveratrol is a promising anti-cancer agent for different breast cancer subtypes with regard to cell apoptosis by inhibiting FASN.

## Data Availability

No new data were created.

## Abbreviations

CCK-8: Cell Counting Kit-8; DMEM, Dulbecco’s modified Eagle’s medium; DMSO, dimethyl sulfoxide; EGFR, epidermal growth factor receptor; FAK, focal adhesion kinase; FASN, fatty acid synthase; FBS, fetal bovine serum; GAPDH, glyceraldehyde-3-phosphate dehydrogenase; IC_50_, half inhibitory concentration; NADPH, nicotinamide adenine dinucleotide phosphate; PA, palmitic acid; PARP, poly(ADP-ribose) polymerase; SDS, sodium dodecyl sulphate; siRNA, small interfering RNA.
